# Muller's Ratchet and compensatory mutation in *Caenorhabditis briggsae *mitochondrial genome evolution

**DOI:** 10.1186/1471-2148-8-62

**Published:** 2008-02-26

**Authors:** Dana K Howe, Dee R Denver

**Affiliations:** 1 Department of Zoology and Center for Genome Research and Biocomputing, Oregon State University, Corvallis, Oregon, 97331, USA

## Abstract

**Background:**

The theory of Muller' Ratchet predicts that small asexual populations are doomed to accumulate ever-increasing deleterious mutation loads as a consequence of the magnified power of genetic drift and mutation that accompanies small population size. Evidence for Muller's Ratchet and knowledge on its underlying molecular mechanisms, however, are lacking for natural populations.

**Results:**

We characterized mitochondrial genome evolutionary processes in *Caenorhabditis briggsae *natural isolates to show that numerous lineages experience a high incidence of nonsynonymous substitutions in protein-coding genes and accumulate unusual deleterious noncoding DNA stretches with associated heteroplasmic function-disrupting genome deletions. Isolate-specific deletion proportions correlated negatively with nematode fecundity, suggesting that these deletions might negatively affect *C. briggsae *fitness. However, putative compensatory mutations were also observed that are predicted to reduce heteroplasmy levels of deleterious deletions. Paradoxically, compensatory mutations were observed in one major intraspecific *C. briggsae *clade where population sizes are estimated to be very small (and selection is predicted to be relatively weak), but not in a second major clade where population size estimates are much larger and selection is expected to be more efficient.

**Conclusion:**

This study provides evidence that the mitochondrial genomes of animals evolving in nature are susceptible to Muller's Ratchet, suggests that context-dependent compensatory mutations can accumulate in small populations, and predicts that Muller's Ratchet can affect fundamental evolutionary forces such as the rate of mutation.

## Background

Evolutionary theory predicts that mutational decay is inevitable for small asexual populations, provided deleterious mutation rates are high enough. Such populations are expected to experience the effects of Muller's Ratchet [1,2] where the most-fit class of individuals is lost at some rate due to chance alone, leaving the second-best class to ultimately suffer the same fate, and so on, leading to a gradual decline in mean fitness. The mutational meltdown theory [3,4] built upon Muller's Ratchet to predict a synergism between mutation and genetic drift in promoting the extinction of small asexual populations that are at the end of a long genomic decay process. Regardless of reproductive mode, mitochondrial genomes from most animal species are expected to be particularly sensitive to Muller's Ratchet due to their uniparental inheritance, high mutation rates and lack of effective recombination [3,5,6]. The genomic decay effects of Muller's Ratchet have been observed in laboratory evolution experiments with abiotic RNA molecules [7], biotic RNA viruses [8], bacteria [9] and yeast [10]. Indirect evidence for the effects of Muller's Ratchet in nature has resulted from studies on the long-term effects of reduced population sizes on genetic diversity and fitness in amphibians [11], greater prairie chickens [12,13] and New Zealand avifauna [14]. Molecular evidence for Muller's Ratchet has resulted from analyses of deleterious tRNA gene structures encoded by mitochondrial genomes [15] and analyses of *Drosophila *sex chromosome evolution [16]. However, direct knowledge on the susceptibilities of natural populations to Muller's Ratchet and the molecular mechanisms underlying this process remain enigmatic.

*Caenorhabditis briggsae*, like *Caenorhabditis elegans*, is a self-reproducing hermaphroditic nematode species that also produces males capable of outcrossing with hermaphrodites. Analyses of linkage disequilibrium patterns in *C. briggsae *natural isolates suggest a very low outcrossing rate of ~3.9 × 10^-5 ^[17]. The same study reported population subdivision between *C. briggsae *strains collected in temperate localities versus those from tropical regions and nuclear silent-site nucleotide diversity (π_S_) for the tropical isolates was estimated at 2.7 × 10^-3 ^– a number highly similar to global estimates for *C. elegans *[18]. The *C. briggsae *isolates from temperate localities, however, showed a remarkably lower mean π_S _value of 4.0 × 10^-5^. A direct estimate of the neutral base substitution mutation rate (9.0 × 10^-9 ^per site per generation) is available from *C. elegans *mutation-accumulation lines [19] that can be used along with π_S _data to estimate the effective population size (*N*_*e*_) [20]. Assuming a common mutation rate between *C. elegans *and *C. briggsae*, *N*_*e *_is estimated to be ~63,000 for *C. briggsae *tropical isolates. For the *C. briggsae *temperate isolates, a much smaller *N*_*e *_of ~1,000 is estimated. Based on these and other observations, it is hypothesized that *C. briggsae *only recently (in the last few hundred years) colonized temperate latitudes from small founding populations [17]. Furthermore, there is evidence for a ~2-fold elevated mutation rate in *C. briggsae *as compared to *C. elegans *[21] that leads to correspondingly lower *N*_*e *_estimates for *C. briggsae*: ~31,500 for tropical populations and ~500 for temperate populations. The combination of very low outcrossing rates, small *N*_*e *_and high mutation rates are expected to render *C. briggsae *natural mitochondrial lineages susceptible to the effects of Muller's Ratchet-associated deleterious mutation accumulation.

To probe for the effects of Muller's Ratchet in *C. briggsae *natural populations, we sequenced nearly complete mitochondrial genomes from multiple geographically diverse *C. briggsae *natural isolates and characterized molecular evolutionary processes by comparing nucleotide diversity patterns in mitochondrial DNA (mtDNA) protein-coding genes between the temperate- and tropical-clade *C. briggsae *isolates, characterizing heteroplasmic genome deletions using quantitative real-time PCR (qPCR) approaches and evaluating correlations of various natural mitochondrial genome haplotypes with nematode fecundity and fitness.

## Results and Discussion

### Noncoding DNA accumulation in *Caenorhabditis *mitochondrial genomes

To search for natural mutational decay in *C. briggsae*, we analyzed nearly complete mitochondrial genome sequences (13,430/14,420 total bp) from 24 geographically diverse *C. briggsae *natural isolates (Table 1). mtDNA sequences were amplified as four overlapping long PCR products (3–5 kb in size each) that were directly sequenced using a combination of PCR and internal sequencing primers (see Methods). A complete mitochondrial genome sequence for *C. briggsae *isolate AF16 is available from Genbank (accession # NC_009885) and was used as a reference for primer design. The mitochondrial genome AT-region constituted the only mtDNA segment not analyzed in the *C. briggsae *natural isolates – as with *C. elegans *[22], efforts to sequence through this highly AT-rich region proved unsuccessful. Each isolate was found to encode a unique mitochondrial genome haplotype.

**Table 1:** *C. briggsae *natural isolate origins and fecundities.

Isolate	Geographic Location	Clade	Fecundity
AF16	Ahmedabad, India	TR	144.5 (5.9)
BW287*	Beijing, China	TE	163.5 (5.7)
ED3032	Taipei, Taiwan	TR	171.8 (9.3)
ED3033	Taipei, Taiwan	TR	111.8 (8.1)
ED3034	Taipei, Taiwan	TR	110.0 (5.0)
ED3035	Taipei, Taiwan	TR	109.0 (8.0)
ED3036	Taipei, Taiwan	TR	98.0 (12.9)
ED3037	Taipei, Taiwan	TR	122.3 (9.5)
ED3083	Johannesburg, S. Africa	TR	100.0 (12.5)
ED3092	Nairobi, Kenya	KE	188.0 (3.3)
ED3101	Nairobi, Kenya	KE	205.8 (8.0)
EG4181	Utah, USA	TE	176.0 (4.3)
EG4207A	Utah, USA	TE	181.0 (4.5)
HK104	Okayama, Japan	TE	162.5 (7.6)
HK105	Sendai, Japan	TE	75.3 (7.2)
JU403	Hermanville, France	TE	105.5 (4.8)
JU439	Reykjavic, Iceland	TE	118.5 (3.3)
JU516	Marsas, France	TE	108.5 (3.7)
JU725	Chengyang, China	TR	167.5 (2.6)
JU726	Tangshuo, China	TR	122.3 (15.7)
JU793	Frechendets, France	TE	132.8 (2.1)
PB800	Ohio, USA	TE	177.5 (10.5)
PB826	Ohio, USA	TE	165.3 (3.0)
VT847	Hawaii, USA	TR	130.0 (6.2)

Strain BW287 (identified by asterisk) has historically been known as a nematode in the genus *Panagrolaimus*; nuclear ribosomal DNA and mtDNA sequencing carried out in the Denver lab, involving strains obtained from three different sources, agree that this isolate is instead a strain of *C. briggsae*. EG4207A was established from a single nematode picked from EG4207 that constituted a *C. briggsae *lab population initially established from many nematodes. For Clade, TE indicates the temperate intraspecific clade, TR the tropical clade and KE the Kenya clade. Fecundity shows the mean numbers of progeny produced for each isolate (four worms assayed per strain) and the S. E. M. is shown in parentheses.

**Figure 1:**
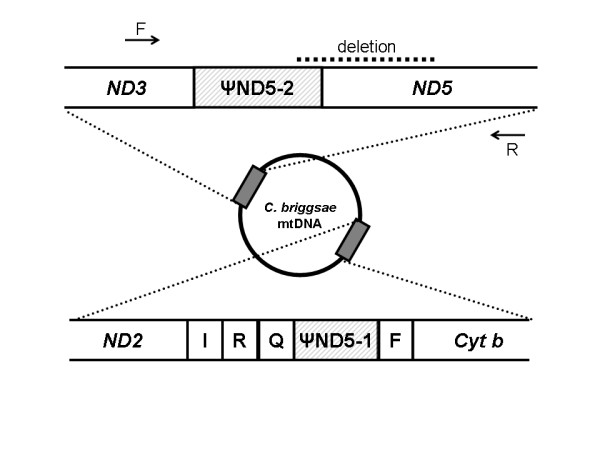
**ψND5 element positions in *C. briggsae *mitochondrial genomes**. Dashed boxes indicate the two ψND5 elements and open boxes indicate mtDNA genes. Protein-coding genes are indicated by their common abbreviations and tRNA genes are indicated by their respective associated single-letter amino acid codes. ψND5-1 is 214–223 bp, depending on isolate, and ψND5-2 is 325–344 bp. The dashed horizontal line on top indicates DNA sequences that are lost in heteroplasmic *ND5 *deletion variants and the arrows indicate the primer positions that are employed for conventional PCR assays.

We discovered that most *C. briggsae *mitochondrial genomes harbor two presumed noncoding regions that are homologous to and likely derive from the NADH dehydrogenase 5 (*ND5*) protein-coding gene. The first *ND5*-like element (named ψND5-1) was identified between two tRNA genes, whereas the second larger element (ψND5-2) resides between the *ND3 *and *ND5 *protein-coding genes (Figure 1). ψND5-1 and ψND5-2 display 89.3% and 88.0% sequence identity, respectively, to homologous regions of the *C. briggsae ND5 *gene. There is 94.1% sequence identity between ψND5-1 and ψND5-2. We subjected the ψND5-1 and ψND5-2 sequences to conceptual translation using the appropriate genetic code in MEGA4 [23] to discover the presence of numerous internal stop codons, suggesting that these elements do not encode functional protein products. Whereas all 24 *C. briggsae *mtDNA sequences analyzed were found to harbor ψND5-1, only 22/24 isolates harbored ψND5-2 – this element was missing from the two Kenya isolate (ED3092, ED3101) mitochondrial genomes [24]. Although the mitochondrial genomes of *C. elegans *and *C. remanei *were found not to encode ψND5 elements, that of *Caenorhabditis *sp. n. 5 (isolate JU727), a recently-identified gonochoristic sister species to *C. briggsae *[25], was discovered to encode a divergent form of ψND5-1 (see Additional file 1).

**Figure 2:**
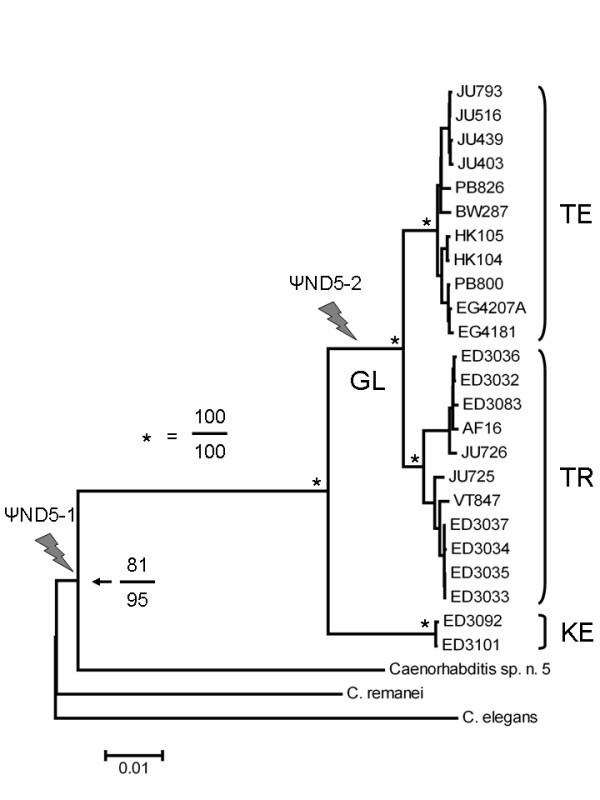
**Evolutionary origins of ψND5 elements**. Intraspecific phylogeny of *C. briggsae *natural isolates based on neighbor-joining analysis of mtDNA sequences is shown. Neighbor-joining and maximum parsimony analyses of nearly complete mtDNA sequences yielded trees with identical topologies. GL indicates the global intraspecific *C. briggsae *superclade; KE indicates the Kenya clade; TE and TR indicate the temperate and tropical subclades of GL, respectively. Bootstrap support of 100% for both analysis approaches was observed for all selected nodes indicated by asterisks – for the node supporting monophyly of *C. briggsae *and *Caenorhabditis *sp. n. 5, neighbor-joining values are on top of maximum parsimony values. The scale bar represents 0.01 substitutions per site. Bolts indicate branches where ψND5 elements are predicted to have originated based on the phylogeny and the presence of ψND5-1 and ψND5-2 in specific *Caenorhabditis *species and strains. The distributions of isolates into temperate and tropical clades in our analyses were consistent with their distributions based on previous analyses of nuclear loci [17].

To investigate the evolutionary relationships of *C. briggsae *natural isolate hermaphrodite lineages and the origins of ψND5-1 and ψND5-2, we subjected the mtDNA sequences to phylogenetic analyses using MEGA4 [23]. Mitochondrial genome sequences from *C. elegans *(accession # NC_001328), *Caenorhabditis remanei *(W. K. Thomas, unpublished data) and *Caenorhabditis *sp. n. 5 (a recently-discovered gonochoristic sister species to *C. briggsae *– sequences collected here for isolate JU727) were used as outgroups. All mtDNA sequences were used for phylogenetic analyses, with the exception of ψND5-1 and ψND5-2 regions since determining their origins was one aim of the phylogenetic studies. Maximum parsimony and neighbor-joining analyses yielded trees with identical topologies. The phylogenies revealed the presence of three major, well-supported intraspecific *C. briggsae *clades that we refer to as the temperate, tropical and Kenya clades (Figure 2). The temperate and tropical clades form a monophyletic superclade, referred to as the global superclade, to the exclusion of the divergent Kenya clade. The phylogenetic distributions of isolates into these clades are highly congruent with previous phylogenetic analyses based on nuclear intron and protein-coding gene sequences [17]. The absence of ψND5-2 in the mitochondrial genomes of the two Kenya clade *C. briggsae *isolates and other *Caenorhabditis *species suggests that this element originated in the lineage leading to the *C. briggsae *global superclade. The presence of ψND5-1 in all *C. briggsae *isolates and *Caenorhabditis *sp. n. 5, but not other *Caenorhabditis *species, suggests that this element originated in the common ancestor of *C. briggsae *and *Caenorhabditis *sp. n. 5 (Figure 2).

With the exception of the AT-region, the vast majority of other animal mitochondrial genomes examined are devoid of similar extensive stretches of noncoding DNA [26]. Based on a recent population-genetic theory on the evolution of genome complexity [27] that predicts higher noncoding DNA accumulation probabilities in species of small population size due to a magnified power of genetic drift, the accumulation and persistence of ψND5 elements in *C. briggsae *mitochondrial genomes is consistent with the hypothesis that natural *C. briggsae *lineages experience very small population sizes that are essential for genomic decay associated with Muller's Ratchet. ψND5-1, however, occurs in both *C. briggsae *and the obligately outcrossing species *Caenorhabditis *sp. n. 5. The ψND5-1 element might have accumulated in the (presumably gonochoristic) ancestor of *C. briggsae *and *Caenorhabditis *sp. n. 5 as a consequence of Muller's Ratchet since nematode mtDNA is inherited through the hermaphrodite/female lineage and is not known to undergo intergenomic recombination. Only two natural isolates from China are presently known and available *Caenorhabditis *sp. n. 5 [28]; thus, we are presently unable to effectively characterize the population-genetic environment in which *Caenorhabditis *sp. n. 5 evolves. Future comparative analyses of ψND5-1 molecular evolution between these two species, however, might provide important insights into the role of outcrossing in eliminating deleterious mutations from natural populations.

### Heteroplasmic mitochondrial genome deletions

**Figure 3:**
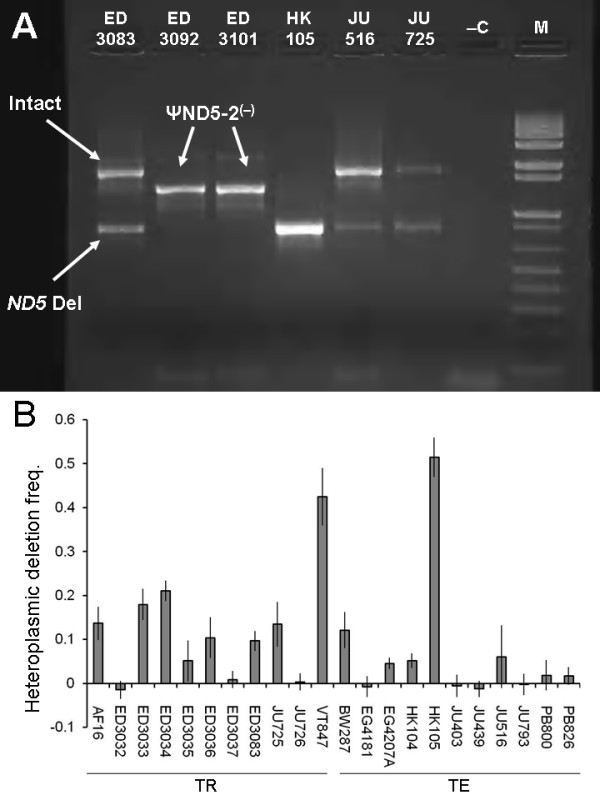
**Heteroplasmic mitochondrial genome deletions**. (A) Amplicons resulting from conventional PCRs with primers flanking the deletion area (primer positions shown in Figure 1) are shown for a subset of isolates analyzed (genomic DNAs were from bulk nematode extracts). Large bands represent intact mitochondrial genomes that contain the ψND5-2 element; small bands represent deletion-bearing genomes. Samples from the two Kenya clade isolates yielded only a single band of intermediate size due to the absence of ψND5-2 in their mitochondrial genomes. -C indicates the negative control (no genomic DNA) and M indicates the molecular marker (1 kb + DNA ladder from Invitrogen). (B) Estimated *ND5 *deletion frequencies (y axis) from 22 global superclade *C. briggsae *natural isolates based on qPCR data. TR indicates tropical-clade isolates and TE indicates temperate-clade isolates. Values shown in bar graph are means from four individual nematodes assayed per genotype – error bars show S. E. M.

Upon further analyzing the ψND5-2 region in the *C. briggsae *natural isolates, we found that some strains harbor a large heteroplasmic mitochondrial genome deletion (871–887 bp, depending on isolate) that eliminated the 3' end of ψND5-2 and the 5' end (first 786 bp) of the *ND5 *protein-coding gene (Figure 1 and 3A). Directly repeated DNA sequence tracts in *ND5 *and the upstream ψND5-2 element flank the heteroplasmic *ND5 *deletion allele and are likely to play a causative role in promoting the deletions as direct repeats are also associated with heteroplasmic mitochondrial DNA deletions in *C. elegans *mutation-accumulations lines [6] and aging nematodes [29]. The observed deletion is expected to strongly and negatively affect *ND5 *protein-coding function as the deleted sequences encode more than 200 *ND5 *amino acids, 34 of which are conserved in *C. elegans*, *Drosophila melanogaster *and humans. The ψND5-2 region and associated heteroplasmic *ND5 *gene deletion was initially discovered in long PCR amplifications of *C. briggsae *natural isolate mitochondrial genome segments. The observation of the heteroplasmic deletion allele across multiple independent long PCR reactions (3 to 5 kb amplicons) that span many mtDNA genes provides strong evidence that the deletion is not a nuclear sequence of mitochondrial origin (numt), but rather a heteroplasmic mtDNA allelic variant. Furthermore, we have searched for the deletion-bearing sequences in the published *C. briggsae *nuclear genome sequence and found no evidence for the presence of a candidate numt. We also found no evidence for heteroplasmic deletions associated with ψND5-1.

To better understand the relative abundances of *ND5 *deletion-bearing and intact mitochondrial genomes among *C. briggsae *natural isolates, we applied both conventional PCR and qPCR approaches. Although initial exploratory studies used bulk DNA extracts from large mixed-stage cultures, our assays were ultimately applied to genomic DNA samples derived from individual L1-stage (first larval stage) nematodes – four independent individuals were assayed per isolate. Initial conventional PCR assays employed a forward primer in the *ND3 *gene (upstream of ψND5-2) and a reverse primer in *ND5 *sequences downstream of the heteroplasmic deletion – genomic DNA samples from most global superclade *C. briggsae *isolates yielded one or two PCR products, one being ~1700 bp (intact genomes) and a second that is ~800 bp (deletion-bearing genomes) – see Figure 3A. DNA samples from the two isolates from Kenya (lack ψND5-2) produced single amplicons of intermediate size, as expected. This PCR reaction was carried out for four independent single-worm genomic DNA samples from each of the 22 global-clade *C. briggsae *natural isolates analyzed using agarose gel electrophoresis; gel banding patterns were scored to evaluate heteroplasmy patterns in their mitochondrial genomes. In particular, gels were scored on the basis of whether amplified products resulted from intact genomes only, deletion-bearing genomes only, or from both intact and deletion-bearing genomes. Substantial PCR product banding pattern variation was observed (see Additional file 2) – among the 88 individual worms assayed, 65 produced only amplicons from intact genomes, 23 yielded amplicons from both intact and deletion-bearing genomes and 3 (all from isolate HK105) exclusively produced amplicons from deletion-bearing genomes.

Although the conventional PCR approach suggested extensive among-isolate variation in deletion heteroplasmy levels, a qPCR approach was applied to provide a more quantitative characterization of variation in the proportion of *ND5 *deletion-bearing genomes to the total among the isolates. qPCRs were carried out using the same genomic DNA samples that were used for the conventional PCR assays (four individuals per isolate). One set of qPCR primers amplified a *ND5 *region present only in intact genomes (5' end of the gene) and a second amplified a 16S rRNA region present in both deletion-bearing and intact genomes for which there is no evidence of heteroplasmy (see Methods). The nematode-specific proportions of genomes bearing *ND5 *deletions were calculated by dividing the estimated abundance of intact genomes by that of the total mitochondrial genomes, and then subtracting that number from one. Estimated deletion-bearing genome proportions from qPCR results correlated positively with conventional PCR band scoring data (Spearman rank correlation = 0.74, *P *< 10^-15^) and revealed substantial among-isolate variation in *ND5 *deletion heteroplasmy levels (Figure 3B). *ND5 *deletion-bearing genomes were observed to accumulate in both the temperate and tropical intraspecific *C. briggsae *clades. Two isolates (HK105, VT847) showed remarkably high deletion genotype proportions greater than 40%. Interestingly, these two isolates were collected from islands (Table 1) where populations might have been recently established from very small populations where drift is expected to play a particularly dominant role [20]. However, other island isolates (e.g. HK104, JU439) displayed low deletion proportions. Although it cannot be ruled out that changes in heteroplasmy levels occurred while nematodes were maintained in laboratory culture after collection from nature, most (at least 19/24) of the *C. briggsae *isolates considered here constitute strains recently collected from the wild [17] that did not spend substantial time in laboratory culture before being stored as cryogenically-preserved frozen stocks. Although similar heteroplasmic mitochondrial genome deletions have been observed in laboratory-bottlenecked *C. elegans *mutation-accumulation lines [6] and are associated with rare mitochondrial myopathies in humans [30], the widespread occurrence and persistence of such deleterious mitochondrial gene deletions in natural populations is unprecedented.

To investigate the potential effects of deletion-bearing molecules on organismal fitness, we next assayed variation in lifetime fecundity in the *C. briggsae *natural isolates. The mean fecundity observed across all 24 *C. briggsae *isolates assayed was 138.6 progeny, a number remarkably smaller than that for *C. elegans *(273.6 progeny [31]), but consistent with previously-reported fitness disparities between these two species [21]. A significantly negative correlation (Pearson's correlation coefficient = -0.41, *P *< 0.05) was observed for isolate-specific deletion genotype proportions and nematode fecundities. Furthermore, the two isolates with the highest mean fecundity values were the two from the Kenya clade (ED3092, ED3101 – see Table 1) that do not encode ψND5-2 or experience associated *ND5 *deletion events. These observations suggest that the accumulation of deletion-bearing mitochondrial genomes might strongly and negatively affect fitness in *C. briggsae *natural populations. The present study, however, is unable to disentangle the fitness effects of mitochondrial and nuclear loci – it is possible that there exist deleterious nuclear loci that are responsible for reduced fitness in these lines rather than the high deletion levels. We expected to observe higher *ND5 *deletion proportions in the temperate-clade *C. briggsae *isolates as compared to those of the tropical clade as a consequence of reduced efficiency of natural selection associated with smaller *N*_*e *_estimates in the former group. However, no significant differences in *ND5 *deletion heteroplasmy levels were observed between these two groups (*P *> 0.9, two-tailed *t*-test).

### Accumulation of putative ψND5-2 compensatory mutations

**Figure 4:**
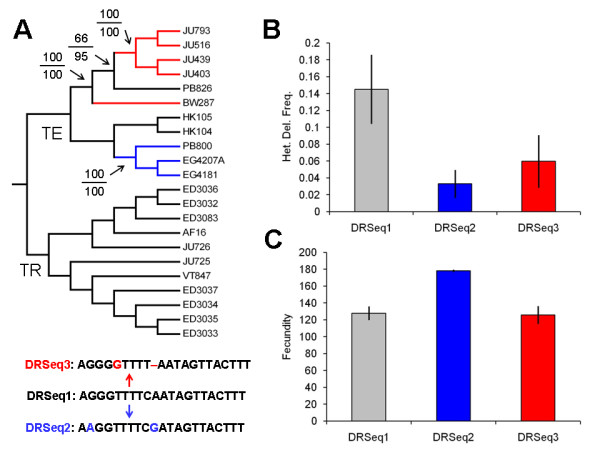
**ψND5-2 compensatory mutations**. (A) Phylogenetic distributions of three ψND5-2 direct repeat sequence haplotypes among temperate and tropical-clade *C. briggsae *isolates are shown on top. Black lines indicate lineages that encode DRSeq1 (identical to downstream *ND5 *sequence), blue lines show lineage with DRSeq2 and red lines show DRSeq3-encoding lineages. Boostrap support (neighbor-joining over maximum parsimony) is shown for relevant nodes. Specific DNA sequence changes in DRSeq2 and DRSeq3 are shown on bottom. (B) Variation in *ND5 *heteroplasmic deletion frequencies among isolates encoding different ψND5-2 sequences is shown. Error bars show S. E. M. (C) Mean fecundities of isolates encoding different ψND5-2 sequences are shown. Error bars show S. E. M.

The heteroplasmic *ND5 *deletion observed in the *C. briggsae *natural isolates is flanked by directly repeated DNA sequence stretches in homologous regions of ψND5-2 and *ND5 *– one repeat copy remains in deletion-bearing genomes. We characterized patterns of sequence divergence at the 21 bp of directly repeated DNA sequence in the *C. briggsae *isolates and discovered the presence of three different haplotype sequences for the repeat unit present in ψND5-2 (Figure 4A); all isolates had identical sequences at the *ND5 *repeat unit. For the ψND5-2 repeat unit, the majority of isolates encoded an ancestral sequence (named DRSeq1 – see Figure 4A) that was identical to that present downstream in *ND5*. In the temperate clade, however, two divergent derived haplotype sequences were observed in the ψND5-2 direct repeat (DRSeq2 and DRSeq3), each containing two sequence differences relative to the downstream *ND5 *repeat, that are expected to render the isolates encoding these divergent sequences less susceptible to direct repeat-associated intragenomic deletion events as compared to the DRSeq1 repeat unit. Consistent with this expectation, isolates that encode DRSeq2 display significantly lower *ND5 *deletion proportions as compared to those that encode DRSeq1 (*P *< 0.05, two-tailed *t*-test) – see Figure 4B. Isolates encoding DRSeq3 also show lower *ND5 *deletion levels, though the difference as compared to those that encode DRSeq1 is not significant (*P *> 0.10, two-tailed *t*-test). When considered as groups, mean heteroplasmic deletion levels were higher in tropical-clade isolates (12.1%) as compared to temperate-clade isolates (7.3%), though the difference was not significant (*P *> 0.9, two-tailed *t*-test). We also analyzed fecundity variation with respect to the three sequence motifs and found that isolates encoding DRSeq2 displayed significantly elevated fecundity as compared to those encoding DRSeq1 (*P *< 0.001, two-tailed *t*-test) – see Figure 4C. Fecundities in isolates encoding DRSeq3 and DRSeq1, however, were highly similar and not significantly different (P > 0.1, two-tailed *t*-test). The relatively low *ND5 *deletion proportions and high fecundities associated with isolates encoding DRSeq2 are suggestive of compensatory mutation – in particular, the ψND5-2 substitutions are expected to result in reduced interactions of directly repeated sequences in ψND5-2 and *ND5*, thereby resulting in lower *ND5 *deletion incidences and higher fitness. However, we are again unable to account for the potential effects of nuclear loci.

These ψND5-2 substitutions that likely promote reduced *ND5 *deletion proportions (DRSeq2, DRSeq3) are observed in the *C. briggsae *intraspecific temperate clade where *N*_*e *_estimates are much lower than that of the tropical clade [17] and, thus, natural selection for compensatory mutations is expected to be less efficient. All tropical clade isolates analyzed encode DRSeq1. However, recent work in phage mutation-accumulation experiments suggests an increase in the relative incidence of beneficial mutations as fitness decreases in association with deleterious mutation accumulation [32]. Our results provide empirical support for this hypothesis in natural animal populations – any mutations in ψND5-2 that result in reduced incidences of direct repeat-induced *ND5 *deletion events are expected to be advantageous. We propose that the occurrence of one highly deleterious mutational event (i.e. the ψND5-2 insertion and associated *ND5 *deletion events) presents a novel mutational target in the mitochondrial genome where most all new mutations are expected to reduce homology between ψND5-2 and *ND5*, thus reducing the likelihood of deleterious mitochondrial genome deletions. Consequently, such new mutations that result in increased divergence between ψND5-2 and *ND5 *can be characterized as advantageous compensatory mutations. The fact that these candidate compensatory mutations were observed in the temperate clade (where *N*_*e *_estimates are relatively small and natural selection is expected to be relatively inefficient) and not the tropical clade (where *N*_*e *_estimates are relatively large and natural selection is expected to be relatively more efficient) is puzzling. Although the evolutionary causes of these putative compensatory mutations in the temperate clade remains mysterious, it is possible that the heteroplasmic deletions are more deleterious in the temperate clade as compared to the tropical clade and that there is a corresponding difference in the selection coefficients associated with the candidate compensatory changes observed in the temperate isolates. Another (probably unlikely) possibility is that the increased fixation probability of these mutations in smaller populations (i.e. temperate-clade isolates) outweighs the higher population mutation rate experienced by larger populations (i.e. tropical-clade isolates). Finally, it is possible that the *N*_*e *_estimates based on nuclear nucleotide diversity data do not accurately reflect the current population-genetic environments experienced by these two intraspecific clades.

After discovery of these putative compensatory mutations, we again searched for elevated *ND5 *deletion levels in the temperate as compared to the tropical isolates, this time including only those temperate-clade isolates that encode DRSeq1. Although the estimated *ND5 *deletion levels were on average greater in DRSeq1-encoding temperate isolates (23.0%) versus that of the tropical isolates (13.1%), the difference was not significant (*P *> 0.1, two-tailed *t*-test). Fecundities were also highly similar (Table 1) and not significantly different (*P *> 0.5, two-tailed *t*-test). Unfortunately, only three of the analyzed temperate-clade isolates encode DRSeq1 (Figure 4), thereby severely limiting our power to detect differences in the propensities of these two intraspecific clades of different *N*_*e *_estimates to accumulate deleterious *ND5 *deletions. Future efforts surveying a greater number of *C. briggsae *natural isolates will be required to further probe for the differential effects of Muller's Ratchet between these two clades at this locus.

### Nonsynonymous substitution accumulation in protein-coding genes

In addition to investigating the evolutionary dynamics of the unusual ψND5 elements, the nearly complete *C. briggsae *mitochondrial genome sequences also offer the opportunity to analyze molecular evolution in the rest of the genome and, in particular, the twelve mtDNA protein-coding genes. The relative efficiency of natural selection in preventing the accumulation of deleterious mutations is often quantified by considering the relative rates of fixed nonsynonymous and synonymous substitutions in protein-coding genes [20,33]. We applied a similar approach to probe for differences between the temperate- (small *N*_*e*_) and tropical-clade (large *N*_*e*_) *C. briggsae *isolates in their propensities for accumulating nonsynonymous substitutions. However, because we are comparing non-fixed nucleotide polymorphism patterns between two intraspecific lineages, we applied the ratio of nucleotide diversity at nonsynonymous sites (π_N_) to that at synonymous sites (π_S_) as a measure of relative susceptibilities to (presumed) deleterious nonsynonymous mutation accumulation, using DnaSP [34]. For ten individual mtDNA protein-coding genes, π_N_/π_S _values were compared between isolates of the *C. briggsae *temperate clade to those of the *C. briggsae *tropical clade. A concatenated data set including codons from all mtDNA protein-coding genes was also analyzed and further compared to a comparable data set from 17 *C. elegans *natural isolates where there is no evidence of global phylogeographic population structure [22].

**Figure 5:**
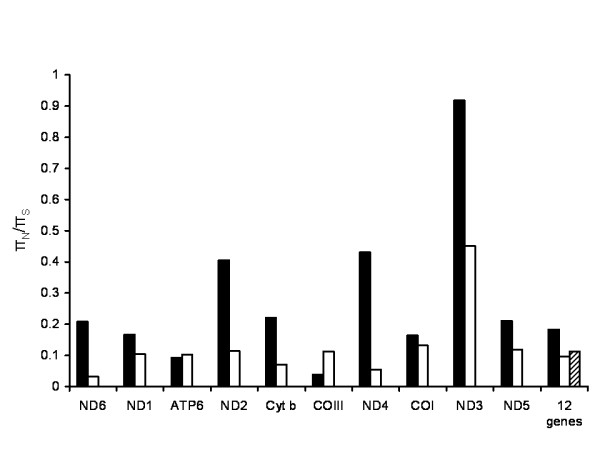
**π_N_/π_S _ratios for mtDNA protein-coding genes**. The π_N_/π_S _ratios for ten mtDNA protein-coding genes were calculated for the temperate-clade (black bars, n = 11) and tropical-clade (white bars, n = 11) *C. briggsae *isolates. Ratios were not calculated for two mtDNA genes because no nonsynonymous substitutions were observed for *ND4L *and *COII *in the temperate and tropical isolates, respectively. Concatenated protein-coding gene data files (12 genes) were also analyzed in the temperate and tropical-clade isolates, as well as in a set of *C. elegans *(dashed bar, n = 17) isolates.

We found that the π_N_/π_S _ratio was greater in the temperate-clade isolates as compared to the tropical-clade isolates for 8/10 genes (Figure 5). For the concatenated gene datasets, the π_N_/π_S _ratio was higher in the temperate-clade *C. briggsae *isolates than that of the tropical-clade *C. briggsae *and *C. elegans *datasets. We attempted to apply meaningful confidence intervals on the π_N _and π_S _estimates using coalescent simulations in DnaSP, however the small numbers of compared sequences (n = 11 for each of the temperate and tropical datasets) and low levels of nucleotide diversity (see Additional file 3) precluded our ability to do so. Nonetheless, we observed dramatic magnitudinal differences in π_N_/π_S _ratios between the temperate- and tropical-clade *C. briggsae *isolates that suggest an increased susceptibility of temperate isolates to the accumulation of nonsynonymous substitutions. The π_N_/π_S _ratio was remarkably high for *ND3 *in the temperate isolates, being nearly equal to one. These observations suggest that *C. briggsae *temperate isolates are particularly susceptible to the accumulation of deleterious mutations throughout the mitochondrial genome, consistent with expectations based on low *N*_*e *_in this intraspecific clade. The specific fitness effects of these nonsynonymous substitutions, however, remain enigmatic and it is possible that some fraction of them (particularly those encoding NADH dehydrogenase complex subunits) might be advantageous in the context of the heteroplasmic *ND5 *deletion products.

## Conclusion

The findings presented here show that in nature *C. briggsae *mitochondrial genomes are susceptible to the accumulation of unusual deleterious mutations that include the insertion of large noncoding DNA stretches (Figures 1 and 2), function-disrupting heteroplasmic genome deletions (Figure 3) and nonsynonymous substitutions in protein-coding genes (Figure 5). This pattern of molecular evolution is consistent with strong roles for mutation and random genetic drift in shaping mitochondrial genome evolution in natural populations of this species. These observations, coupled with the strong potential negative impact of heteroplasmic *ND5 *deletions on organismal fitness, also suggest that *C. briggsae *mitochondrial genomes evolving in nature are vulnerable to the effects of Muller's Ratchet. Although we did observe higher π_N_/π_S _ratios for protein-coding genes in the temperate (small *N*_*e *_estimate) versus tropical (larger *N*_*e *_estimate) isolates, we were unable to detect a role for Muller's Ratchet in promoting higher deleterious *ND5 *deletion levels in the temperate clade. However, we also observed the accumulation of putative compensatory mutations (DRSeq2) in temperate-clade *C. briggsae *lineages (Figure 4) that likely reduce *ND5 *deletion levels. The DRSeq2 mutations can only be considered beneficial, however, in the context of the preexisting deleterious ψND5-2 insertion mutation – in fact, virtually any mutation in ψND5-2 that reduces its homology to *ND5 *is likely to be beneficial to some extent.

**Figure 6:**
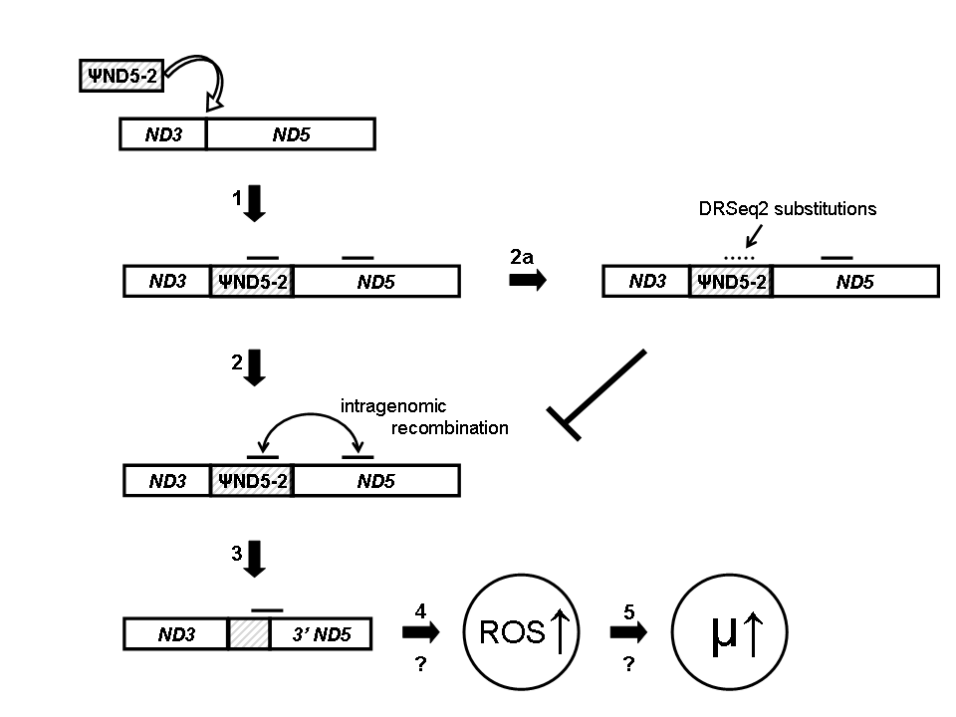
**Summary of proposed molecular evolutionary causes and consequences of heteroplasmic *ND5 *deletions**. The insertion of ψND5-2 (arrow 1) in the ancestor to the global *C. briggsae *clade (see Figure 2) is hypothesized to result in direct repeat-induced intragenomic recombination events (arrow 2) that in turn lead to heteroplasmic *ND5 *gene deletions (arrow 3). Expression of truncated *ND5 *gene products is hypothesized to promote elevated reactive oxygen species levels (arrow 4) that in turn promote higher mutation rates (arrow 5). Accumulation of substitutions (e.g. DRSeq2) in ψND5-2 direct repeats (arrow 2a) are hypothesized to cause reduced intragenomic recombination rates, thereby preventing the elevation of reactive oxygen species levels and associated mutation rate increases.

Comparative nematode mutation-accumulation studies show a significantly elevated deleterious genomic mutation rate in bottlenecked lines derived from *C. briggsae *versus *C. elegans *progenitors [18]. Furthermore, an elevated deleterious mutation rate was estimated for *C. briggsae *HK104-derived lines (encode DRSeq1) as compared to MA lines derived from the PB800 isolate that encodes DRSeq2, the candidate compensatory mutation that likely reduced *ND5 *deletion product levels [21]. The protein product encoded by the *ND5 *gene, predicted to be highly impaired in *ND5 *deletion-bearing genome products, is a central core subunit of Complex I of the mitochondrial electron transport chain [35]. Complex I-compromised organisms in well-studied animal systems- mammals, *D. melanogaster*, and *C. elegans *[36-38] – all produce elevated levels of mutagenic reactive oxygen species. Thus, we predict that *C. briggsae ND5 *deletion products are mutagenic, and that expression of truncated *ND5 *protein products results in elevated reactive oxygen species levels. One intriguing hypothesis for the mutational disparities observed between *C. elegans *and *C. briggsae*, and between HK104 and PB800 (note the close evolutionary relationships between these isolates in Figure 2), is that the elevated rates are due to increased mutagenic reactive oxygen species levels resulting from *ND5 *deletion products (Figure 6). This hypothesis also predicts that Muller's Ratchet can affect fundamental evolutionary parameters such as the mutation rate.

## Methods

### PCR, DNA sequencing and phylogenetics

mtDNA sequencing and PCR were performed as previously described [6,22], with the exception that mitochondrial genome sequences were initially amplified as four overlapping PCR products 3–5 kb in size each using the Expand Long Range PCR kit (Roche). e2TAK (Takara) proofreading DNA polymerase was used for all conventional PCRs. For single-worm DNA extractions, individual worms were picked at the L1 larval stage and digested in 18 μL of lysis buffer [6,22]. 1 μL was then used for each PCR. In *C. elegans*, L1-stage nematodes are estimated to harbor ~25,000 mitochondrial genomes [39]. MEGA4 was used for sequence alignments and phylogenetic analyses (both neighbor-joining and maximum parsimony approaches were applied) [23]. The maximum-composite likelihood molecular evolution model was used for neighbor-joining analysis. 1,000 bootstrap replicates were performed for all phylogenetic statistical testing. Primer sequences used for PCR and DNA sequencing are available upon request. DNA sequences generated for this study were submitted to Genbank under accession numbers EU407780–EU407805.

### qPCR heteroplasmy analysis

qPCR was carried out on an Applied Biosystems 7300 Real Time PCR machine using iTaq SYBR Green Supermix with ROX (Bio-Rad). 1 uL of genomic DNA (diluted 1:5 in water) was used for each qPCR analysis. To estimate the total amount of mtDNA in a sample (both intact and deletion-bearing), control primers were designed to amplify a 102 bp region of the small ribosomal RNA subunit that displayed no evidence of heteroplasmy. A second set of primers was designed to amplify 101 bp in the 5' end of the *ND5 *gene, within the deleted region. This product is not expected to amplify only in intact genomes. qPCR data were analyzed to estimate the abundances of the two genome types using the linear regression approach offered by the LinRegPCR software [40]. *ND5 *locus products were found to amplify more efficiently than ribosomal RNA products; thus, all *ND5 *qPCR values were normalized to account for this disparity. Individual deletion genotype proportions were calculated by dividing the estimated abundance of intact genomes by that of the total mitochondrial genomes, and then subtracting that number from one. Four individual L1-stage nematodes were analyzed per natural isolate – the same nematode genomic DNA samples used for conventional PCR assays.

### Fecundity assay

*C. briggsae *isolate-specific fecundities were estimated by counting the numbers of progeny produced by individual hermaphrodite worms using standard methods involving Toluidine dye-stained plates [21]. The fecundity assay was carried out on standard OP50 *Escherichia coli*-seeded NGM agar plates at 20°C. Progeny production was measured for four independent nematodes per isolate. Prior to testing for relationships between fecundity and qPCR data using the Pearson's correlation approach, both data sets were confirmed to fit a normal distribution using the Kolmogorov-Smirnov test for continuous variables.

### Nucleotide diversity analysis

Aligned mtDNA protein-coding gene sequences were subjected to nucleotide diversity analyses (π_N_/π_S_) using DnaSP v4.0 [34]. The genetic code in DnaSP was set to that for flatworm mtDNA which is identical to that for nematode mtDNA [41]. π_N _and π_S _were individually calculated for each of the twelve mtDNA protein-coding genes in addition to a concatenated data file that contained all mtDNA protein-coding gene codons – 3,414 total. A *C. elegans *concatenated mtDNA file (n = 2,785 codons) was also analyzed – data derived from a previous study [22] and two newly-sequenced genomes from strain JU258 (Madeira Islands, Portugal) and PS2025 (Altadena, CA, USA). Estimates of nucleotide diversity based on theta and the Jukes-Cantor method of calculating π [34] each yielded highly similar results to those presented in Figure 5. Additional file 3 reports individual π_N _and π_S _values.

## Abbreviations

mtDNA: mitochondrial DNA; *N*_*e*_: effective population size; numt: nuclear sequence of mitochondrial origin; qPCR: quantitative real-time PCR; π_N_: nonsynonymous-site nucleotide diversity; π_S_: synonymous-site nucleotide diversity

## Authors' contributions

DKH and DRD designed the study, performed the experiments, analyzed the data and wrote the paper. DRD conceived the study. All authors read and approved the final manuscript.

## Supplementary Material

Additional File 1 **Alignment of *ND5 *and ψND5-1 elements from *C. briggsae *and *Caenorhabditis *sp. n. 5**. This figure shows alignments of *ND5 *and ψND5-1 that were performed using ClustalW (gap parameters set to default: open = 10, extend = 5); residues conserved in all four sequences are in red. The sequence from isolate AF16 was used to represent *C. briggsae *and sequence from isolates JU727 represented *Caenorhabditis *sp. n. 5. The entire ψND5-1 sequences were input into the alignment program whereas input *ND5 *sequences included only the first 990 bp. As with *C. briggsae*, the ψND5-1 element in *Caenorhabditis *sp. n. 5 is located between the tRNA^Q ^and tRNA^F ^genes. Click here for file

Additional File 2 ***ND5 *deletion heteroplasmy data**. This supplementary table provides conventional PCR band scoring data and qPCR results for *ND5 *deletion heteroplasmy analyses. Click here for file

Additional File 3 **π**_N_**and π**_S_**values for mtDNA protein-coding genes**. This supplementary table reports individual nucleotide diversity estimates used to calculate π_N_/π_S _ratios. Click here for file
