# Supplementary figures and images for: Muller's Ratchet and compensatory mutation in Caenorhabditis briggsae mitochondrial genome evolution
Source: BMC Evol Biol. 2008 Feb 26;8:62. doi: 10.1186/1471-2148-8-62 (PMC2279117; doi:10.1186/1471-2148-8-62)

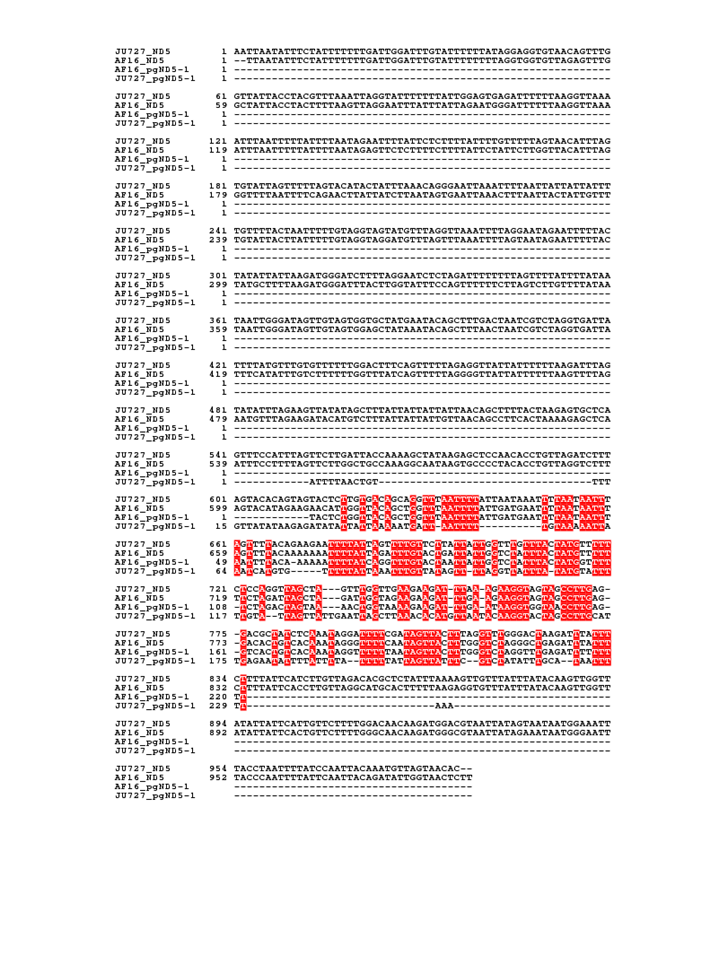

Supplement: Additional File 1 — Alignment of ND5 and ψND5-1 elements from C. briggsae and Caenorhabditis sp. n. 5. This figure shows alignments of ND5 and ψND5-1 that were performed using ClustalW (gap parameters set to default: open = 10, extend = 5); residues conserved in all four sequences are in red. The sequence from isolate AF16 was used to represent C. briggsae and sequence from isolates JU727 represented Caenorhabditis sp. n. 5. The entire ψND5-1 sequences were input into the alignment program whereas input ND5 sequences included only the first 990 bp. As with C. briggsae, the ψND5-1 element in Caenorhabditis sp. n. 5 is located between the tRNAQ and tRNAF genes. [file 1471-2148-8-62-S1.TIFF]
