# Supplementary material for: Muller's Ratchet and compensatory mutation in Caenorhabditis briggsae mitochondrial genome evolution
Source: BMC Evol Biol. 2008 Feb 26;8:62. doi: 10.1186/1471-2148-8-62 (PMC2279117; doi:10.1186/1471-2148-8-62)
Supplement: Additional File 2 — ND5 deletion heteroplasmy data. This supplementary table provides conventional PCR band scoring data and qPCR results for ND5 deletion heteroplasmy analyses. [file 1471-2148-8-62-S2.DOC]

| ***Supplementary table 1 – qPCR and conventional PCR* ND5 *heteroplasmy data.*** | | | |
| --- | --- | --- | --- |
| Genotype | Rep. | qPCR Del Prop. | cPCR Band Score |
|  |  |  |  |
| AF16 | 1 | 0.27 | 2 |
| AF16 | 2 | 0.19 | 1 |
| AF16 | 3 | 0.16 | 1 |
| AF16 | 4 | -0.08 | 1 |
| BW287 | 1 | 0.32 | 2 |
| BW287 | 2 | 0.17 | 2 |
| BW287 | 3 | 0.07 | 1 |
| BW287 | 4 | -0.07 | 1 |
| ED3032 | 1 | 0.07 | 1 |
| ED3032 | 2 | 0.04 | 1 |
| ED3032 | 3 | -0.08 | 1 |
| ED3032 | 4 | -0.09 | 1 |
| ED3033 | 1 | 0.26 | 2 |
| ED3033 | 2 | 0.28 | 2 |
| ED3033 | 3 | 0.21 | 1 |
| ED3033 | 4 | -0.03 | 1 |
| ED3034 | 1 | 0.09 | 1 |
| ED3034 | 2 | 0.18 | 1 |
| ED3034 | 3 | 0.26 | 1 |
| ED3034 | 4 | 0.30 | 2 |
| ED3035 | 1 | -0.11 | 1 |
| ED3035 | 2 | 0.10 | 1 |
| ED3035 | 3 | 0.29 | 2 |
| ED3035 | 4 | -0.08 | 1 |
| ED3036 | 1 | 0.38 | 2 |
| ED3036 | 2 | -0.03 | 1 |
| ED3036 | 3 | 0.07 | 1 |
| ED3036 | 4 | 0.00 | 1 |
| ED3037 | 1 | -0.06 | 1 |
| ED3037 | 2 | -0.05 | 1 |
| ED3037 | 3 | 0.03 | 1 |
| ED3037 | 4 | 0.11 | 1 |
| ED3083 | 1 | 0.01 | 1 |
| ED3083 | 2 | 0.06 | 1 |
| ED3083 | 3 | 0.10 | 1 |
| ED3083 | 4 | 0.22 | 2 |
| EG4181 | 1 | -0.02 | 1 |
| EG4181 | 2 | 0.13 | 1 |
| EG4181 | 3 | -0.05 | 1 |
| EG4181 | 4 | -0.09 | 1 |
| EG4207A | 1 | 0.07 | 1 |
| EG4207A | 2 | 0.00 | 1 |
| EG4207A | 3 | 0.00 | 1 |
| EG4207A | 4 | 0.11 | 2 |
| HK104 | 1 | 0.14 | 2 |
| HK104 | 2 | 0.00 | 1 |
| HK104 | 3 | 0.00 | 1 |
| HK104 | 4 | 0.06 | 1 |
| HK105 | 1 | 0.26 | 2 |
| HK105 | 2 | 0.68 | 3 |
| HK105 | 3 | 0.54 | 3 |
| HK105 | 4 | 0.58 | 3 |
| JU403 | 1 | 0.03 | 1 |
| JU403 | 2 | -0.03 | 1 |
| JU403 | 3 | -0.13 | 1 |
| JU403 | 4 | 0.11 | 1 |
| JU439 | 1 | -0.01 | 1 |
| JU439 | 2 | -0.07 | 1 |
| JU439 | 3 | 0.09 | 1 |
| JU439 | 4 | -0.06 | 1 |
| JU516 | 1 | 0.42 | 2 |
| JU516 | 2 | 0.11 | 1 |
| JU516 | 3 | -0.26 | 1 |
| JU516 | 4 | -0.03 | 1 |
| JU725 | 1 | 0.41 | 2 |
| JU725 | 2 | -0.04 | 1 |
| JU725 | 3 | 0.17 | 2 |
| JU725 | 4 | 0.00 | 1 |
| JU726 | 1 | 0.10 | 1 |
| JU726 | 2 | -0.09 | 1 |
| JU726 | 3 | 0.01 | 1 |
| JU726 | 4 | -0.01 | 1 |
| JU793 | 1 | -0.14 | 1 |
| JU793 | 2 | 0.00 | 1 |
| JU793 | 3 | 0.05 | 1 |
| JU793 | 4 | 0.08 | 1 |
| PB800 | 1 | -0.03 | 1 |
| PB800 | 2 | -0.11 | 1 |
| PB800 | 3 | 0.21 | 2 |
| PB800 | 4 | 0.00 | 1 |
| PB826 | 1 | 0.03 | 1 |
| PB826 | 2 | -0.10 | 1 |
| PB826 | 3 | 0.08 | 1 |
| PB826 | 4 | 0.06 | 1 |
| VT847 | 1 | 0.57 | 2 |
| VT847 | 2 | 0.11 | 2 |
| VT847 | 3 | 0.31 | 2 |
| VT847 | 4 | 0.70 | 2 |
|  |  |  |  |
| **DNA extractions were carried out for four individual nematodes (Rep.) to assay *ND5* deletion heteroplasmy patterns using both qPCR and cPCR approaches. qPCR Del. Prop. indicates the qPCR-estimated *ND5* deletion genotype proportion for each worm (see Materials and Methods). cPCR Band Score indicates the banding pattern observed in conventional PCR products resulting from amplifications spanning the heteroplasmic deletion sequences: 1 = large bands (intact genomes) only observed; 2 = large and small bands (intact and deletion-bearing genomes) observed; 3 = small bands (deletion-bearing genomes) only observed.** | | | |
