# Supplementary material for: Muller's Ratchet and compensatory mutation in Caenorhabditis briggsae mitochondrial genome evolution
Source: BMC Evol Biol. 2008 Feb 26;8:62. doi: 10.1186/1471-2148-8-62 (PMC2279117; doi:10.1186/1471-2148-8-62)
Supplement: Additional File 3 — πNand πSvalues for mtDNA protein-coding genes. This supplementary table reports individual nucleotide diversity estimates used to calculate πN/πS ratios. [file 1471-2148-8-62-S3.DOC]

| ***Supplementary table 2 – π_N_ and π_S_ values for mtDNA protein-coding genes.*** | | | | |
| --- | --- | --- | --- | --- |
| Locus | TE π_N_ | TE π_S_ | TR π_N_ | TR π_S_ |
| ND6 | 0.00199 | 0.00957 | 0.00055 | 0.01759 |
| ND4L | ND | 0.02211 | 0.00197 | 0.02216 |
| ND1 | 0.00201 | 0.01203 | 0.00354 | 0.01203 |
| ATP6 | 0.0004 | 0.00434 | 0.00233 | 0.02275 |
| ND2 | 0.00307 | 0.00757 | 0.00224 | 0.01971 |
| Cyt b | 0.00335 | 0.01516 | 0.00206 | 0.02904 |
| COIII | 0.00074 | 0.01919 | 0.00274 | 0.0242 |
| ND4 | 0.0013 | 0.00302 | 0.00098 | 0.01806 |
| COI | 0.00093 | 0.00569 | 0.00182 | 0.01386 |
| COII | 0.00103 | 0.01368 | ND | 0.02773 |
| ND3 | 0.00424 | 0.00462 | 0.00422 | 0.00937 |
| ND5 | 0.00198 | 0.0094 | 0.00256 | 0.02163 |
| 12 genes | 0.00175 | 0.00955 | 0.00212 | 0.02193 |
| **For the *C. elegans* concatenated 12-gene data set, π_N_ = 0.00286 and π_S_ = 0.02551. ND indicates not determined – no synonymous substitiutions present.** | | | | |
